# Measurement characteristics of the childhood Asthma-Control Test and a shortened, child-only version

**DOI:** 10.1038/npjpcrm.2016.75

**Published:** 2016-10-20

**Authors:** Christian Bime, Joe K Gerald, Christine Y Wei, Janet T Holbrook, William G Teague, Robert A Wise, Lynn B Gerald

**Affiliations:** 1 Division of Pulmonary, Critical Care, Allergy, and Sleep Medicine, Department of Medicine, University of Arizona School of Medicine, Arizona Respiratory Center, Tucson, AZ, USA; 2 Mel and Enid Zuckerman College of Public Health, Department of Health Promotion Sciences, University of Arizona, Arizona Respiratory Center, Tucson, AZ, USA; 3 Department of Epidemiology, Johns Hopkins Bloomberg School of Public Health, Baltimore, MD, USA; 4 Department of Pediatrics, University of Virginia School of Medicine, Charlottesville, VA, USA; 5 Division of Pulmonary, Critical Care, Allergy, and Sleep Medicine, Department of Medicine, Johns Hopkins School of Medicine, Baltimore, MD, USA

## Abstract

The childhood Asthma-Control Test (C-ACT) is validated for assessing asthma control in paediatric asthma. Among children aged 4–11 years, the C-ACT requires the simultaneous presence of both parent and child. There is an unmet need for a tool that can be used to assess asthma control in children when parents or caregivers are not present such as in the school setting. We assessed the psychometric properties and estimated the minimally important difference (MID) of the C-ACT and a modified version, comprising only the child responses (C-ACTc). Asthma patients aged 6–11 years (*n*=161) from a previously completed multicenter randomised trial were included. Demographic information, spirometry and questionnaire scores were obtained at baseline and during follow-up. Participants or their guardians kept a daily asthma diary. Internal consistency reliabilities of the C-ACT and C-ACTc were 0.76 and 0.67 (Cronbach’s *α*), respectively. Test–retest reliabilities of the C-ACT and C-ACTc were 0.72 and 0.66 (intra-class correlation), respectively. Significant correlations were noted between C-ACT scores and ACQ scores (Spearman’s correlation *r*=−0.56, 95% CI (−0.66, −0.44), *P*<0.001). The strength of the correlation between C-ACTc scores and ACQ scores was weaker (Spearman’s correlation *r*=−0.46, 95% CI (−0.58, −0.33), *P*<0.001). We estimated the MID for the C-ACT and C-ACTc to be 2 points and 1 point, respectively. Among asthma patients aged 6–11 years, the C-ACT had good psychometric properties. The psychometric properties of a shortened child-only version (C-ACTc), although acceptable, are not as strong.

## Introduction

One of the main goals of asthma therapy is to achieve and maintain good asthma control.^1^ Asthma control is best assessed using patient-reported outcomes.^2^ Unfortunately, there are a few validated instruments for use in paediatric populations. Among children of 5–11 years of age, the only measure of asthma control recommended by the 2010 National Institutes of Health (NIH) Asthma Outcomes Workshop is the childhood Asthma Control Test (C-ACT).^3^ The C-ACT is well validated for use among children aged 4–11 years.^4^ It is comprised of 3 parent-reported and 4 child-reported items and thus requires the simultaneous presence of both parent and child. There is need for an instrument that is suitable for use in settings where parents or caregivers are not readily accessible or may not be familiar with the child’s perception of disease burden.^5^ One important setting is in schools.^6–10^ Schools are often tasked with monitoring and assessing asthma in children, but there are no readily available tools for assessing asthma control.^8–10^ Such a tool may also be useful in a clinic setting when the guardian who accompanies the child to the visit might not have detailed knowledge about the child’s asthma. For example, many children spend time in several homes (mother, father, grandparents etc.) and may spend significant time away from home (school, daycare and after-school care). Therefore, the accompanying guardian may not have sufficient information to accurately complete asthma control questionnaires. At present, there are no validated alternatives to assess asthma control in children of 4–11 years of age in the absence of the parent or caregiver. Asthma diaries could serve as an alternative, especially if administered as a web-based diary.^11,12^ However, use of asthma diaries among younger patients in the absence of parents has not been demonstrated.

We examined the psychometric properties of the child responses (C-ACTc) and the full C-ACT, among paediatric patients enrolled in a multicenter clinical trial addressing the use of Lansoprazole in children with poor asthma control and without symptomatic gastroesophageal reflux (GER; NCT00442013).^13^ We also obtained a preliminary estimate of the minimally important difference (MID) for the C-ACT and C-ACTc.

## Results

### Study population

Data from 161 study participants aged 6–11 years from the SARCA trial were included (Table 1). The mean age of these participants was 9 years (s.d., 1.6). A majority were male (63%) and black (50%). Twenty-eight per cent and 18% of the participants were white and Hispanic, respectively. At baseline, 51% of study participants reported using combination inhaled corticosteroid/long-acting β_2_ treatment within the past 6 months and 74% reported use of systemic corticosteroids for asthma within the past year.

### Reliability

At baseline, the internal consistency reliability (Cronbach’s *α*) was 0.76 for the C-ACT and 0.67 for the C-ACTc. The test–retest reliability (ICC) for C-ACT scores between two consecutive visits among subjects with stable asthma ranged from 0.44 to 0.94, with an average of 0.72 (Table 2). For the C-ACTc, the range was 0.37 to 0.88, with an average of 0.66. ICCs for both C-ACT and C-ACTc improved over time.

### Construct validity

For the C-ACT, statistically significant Spearman’s correlations were observed between baseline C-ACT scores and baseline ACQ, baseline ASUI scores and pAQLQ. For the C-ACTc, statistically significant Spearman’s correlations were also observed between baseline C-ACTc scores and other asthma questionnaires: ACQ, ASUI and pAQLQ (Table 3).

### Responsiveness

Mean C-ACT scores were significantly lower, indicating worse asthma control, among participants who experienced an episodes of poor asthma control (EPAC) when compared with those who did not (Table 4). The mean difference in C-ACT score between participants with an EPAC versus those without an EPAC was 1.6 points (95% CI (1.0, 2.0), *P*<0.001). Similarly, the mean C-ACTc scores were significantly lower, among patients who had experienced an EPAC when compared with those who did not (Table 5). The mean difference in C-ACTc score between participants with an EPAC versus those without an EPAC was 0.38 points (95% CI (0.14, 0.63), *P*<0.01).

The C-ACT and C-ACTC scores distinguished between groups with good control versus those with poor control. As hypothesised, C-ACT and C-ACTc scores improved among participants who experienced an improvement in asthma control. For those who experienced worsening control, the C-ACT and C-ACTc scores decreased. However, these changes were not statistically significant for the C-ACTc. (Table 6 and Supplementary Table 1).

### MID

We used two distribution-based approaches to estimate the MID for the C-ACT and C-ACTc.^14,15^ Therefore, based on the s.d. of 4.1, and the s.e.m. of 2 for the C-ACT in our study population, we estimated the MID for the C-ACT to be 2 points. Using the anchor-based approach, we determined a mean difference in C-ACT scores between visits with an EPAC in the prior period and visits without an EPAC of 1.56 points. By rounding up to the nearest digit, the MID for the C-ACT was estimated to be 2 points. In adults, the MID for the ACT questionnaire has been estimated to be 3 points.^16^

The s.d. and s.e.m. of the C-ACTc were 2.2 and 1.26, respectively. By using the distribution-based approach, we estimated the MID for the C-ACTc in our study population to be 1 point. With the anchor-based approach, we determined the mean difference in C-ACTc scores between visits with an EPAC in the prior period and visits without an EPAC to equal 0.56 points. By rounding up to the nearest digit, we estimated the MID for the C-ACTc at 1 point.

## Discussion

### Main findings

The main findings of this study are that the C-ACT questionnaire has good psychometric properties in a population of paediatric patients with poorly controlled asthma. The psychometric properties of a shortened version with only the responses of the child, the C-ACTc, although acceptable, are not as good. We estimated the MID for the C-ACT and the C-ACTc to be 2 points and 1 point, respectively. The estimate of MID for the C-ACTc is a relatively larger change and needs further validation. The reliability coefficient for the C-ACTc was 0.67 compared with 0.76 for the C-ACT. Generally, values of 0.70–0.95 are considered acceptable; however, the observed lower *α* could be because of the shorter number of questions in the C-ACTc. At a group level, both questionnaires distinguished between patients with poor control versus those with good asthma control. We also found that for both the C-ACT and C-ACTc, ICCs improve over time which suggests that there may be a learning curve for the children. However, this does not affect the interpretation of the psychometric properties as both questionnaires showed responsiveness to changes in asthma control.

### Strengths and limitations of this study

The strengths of this analysis include the following: the use of robust statistical methods to assess the psychometric properties and to estimate the minimal important difference of the C-ACT and C-ACTc; and the inclusion of a group of well-characterised patients who were prospectively recruited for an asthma intervention study. However, there are several notable limitations. First, in assessing the shortened version (C-ACTc), we recognise that even though the C-ACT instructions indicate that the parents should not help the child complete their section of the questionnaire, it is possible that some of the child responses might be influenced by the parent or caregiver present. Second, we demonstrated an ability to differentiate people with good and bad control based on EPAC analysis but did not demonstrate responsiveness. The C-ACT looked like it could capture improvement but not decline within the range of change that occurred in this cohort. Responsiveness to change at the individual level is best assessed with a prospective design using provider assessment of change in asthma control as an anchor. Therefore, in this *post hoc* analysis of the data collected as part of a randomised control trial, we were unable to assess responsiveness to change in C-ACT and C-ACTc scores at the individual level. In addition, our study population was restricted to patients with poorly controlled asthma. Without a full spectrum of disease severity, it is difficult to accurately assess responsiveness at the individual level. Another limitation of this study is the use of EPACs as anchors to determine the MID of the C-ACT. We did not have data on other anchors such as physician’s global rating of change in asthma control or the patient’s subjective assessment of change in asthma control over time. However, information about EPACs was prospectively collected with asthma diaries. The external validity of the study to children with mild or controlled asthma is limited because only patients with poorly controlled asthma were included and also because our study population had a relatively high representation of African–American children (50%).

### Interpretation of findings in relation to previously published work

Liu *et al*.^4^ have previously shown that C-ACT scores have good internal consistency reliability, test–retest reliability and construct validity in paediatric asthma. Asthma is characterised by significant variations in daily symptoms, and the data on asthma morbidity can be captured using daily asthma diaries. However, asthma diaries are burdensome, especially for children. The ability to recall and discriminate differences in asthma symptoms is also likely to be highly variable among children. One study compared daily diary report of symptoms with the Pediatric Asthma Health Outcome Measure (PAHOM)^17^ questionnaire recall among elementary school children and found poor agreement after two days.^18^ Children underreported asthma symptoms on the PAHOM questionnaire when compared with those in diary entries.^18^ Other studies have confirmed the weak agreement between parent and child responses to asthma questionnaires.^5,19–23^ Inclusion of only the child-reported items in the C-ACT questionnaire is meant to measure the disease burden from the child’s point of view.

### Implications for future research, policy and practice

There is an unmet need for a tool that can be used to assess asthma control in children when parents or caregivers are not present, such as in the school setting or in a clinical setting, as there are times when the guardian who accompanies the child to the visit might not have much detailed knowledge about the child’s asthma. The C-ACTc is therefore a promising instrument for assessing asthma control in these settings. We recognise that the child-reported items were originally designed as part of the C-ACT questionnaire and not intended to be scored separately. However, possible improvements to validating the C-ACTc would include: review of the face validity, a prospective study design with provider and parent rating of change in asthma control as an anchor, including asthma patients with a broad spectrum of asthma severity and control, and making sure that the questionnaire is administered to the child in the absence of the parent or caregiver as would be the case in a school setting. Alternatively, an instrument to meet this need could be developed from the start with item generation, selection, reduction and validation. Although this might delay the availability of an acceptable child-only instrument, it may be possible to improve upon general test characteristic of the instrument.

### Conclusions

In summary, we showed that among asthma patients aged 6–11 years, the C-ACT has good psychometric properties and a shortened version with only the responses of the child, the C-ACTc is promising but needs additional study before it can be used in scientific research and daily practice. We also estimated the MID for the C-ACT and C-ACTc in our study population to be 2 points and 1 point, respectively.

## Materials and Methods

### Data collection

#### Patients

Data from 161 asthma patients 6–11 years of age, enrolled in the Study of Acid Reflux in Children with Asthma (SARCA) clinical trial (NCT00442013) were included in this analysis.^13^ The SARCA clinical trial was conducted by the American Lung Association Asthma Clinical Research Centers (ALA-ACRC) network from April 2007 to September 2011. Among other exclusion criteria, patients were also excluded if they had a history of neonatal respiratory distress or premature birth at less than 33 weeks' gestational age, had a forced expiratory volume in the first second (FEV_1_) of less than 60% predicted, or were non-adherent (<80% completion of daily diaries during run-in).^13^ Prior to enrolment, all study participants were treated with inhaled corticosteroids (⩾176 μg d^−1^ of fluticasone equivalents) and had no change in controller therapies for at least 8 weeks.^13^

#### Procedures

**Figure 1:**
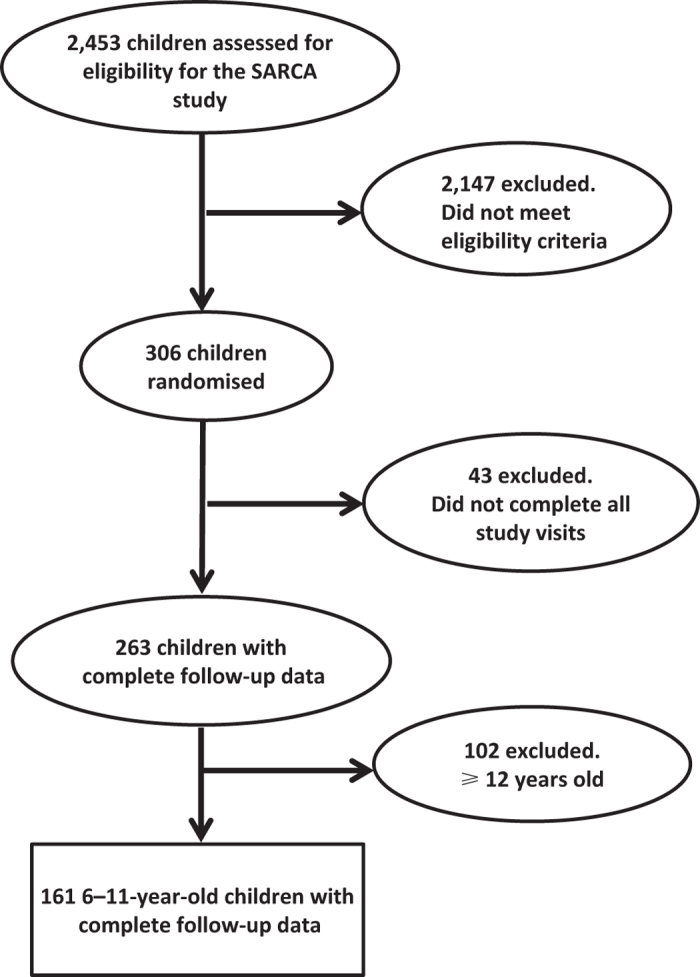
Participant flow.

In the SARCA trial (*N*=306), baseline demographic data, spirometry, Asthma Control Questionnaire (ACQ)^24^ score, C-ACT score (ages 6–11 years), ACT score (ages 12–17 years), Asthma Symptom Utility Index (ASUI)^25^ score and the Pediatric Asthma Quality of Life Questionnaire (pAQLQ)^26^ score were obtained. It should be noted that participants were enrolled for the study only if they had an ACQ score of 1.25 or higher. They were then randomised to either lansoprazole (15 mg per day for children weighing <30 kg; 30 mg per day for children weighing ⩾30 kg) or matching placebo in addition to their inhaled corticosteroids. Participants returned to clinical centres every 4 weeks for evaluation that included spirometry and questionnaire scores. Participants also completed a daily dairy documenting daily morning peak expiratory flow, daily asthma symptoms scores, β_2_ use, nocturnal asthma awakenings, asthma treatments and healthcare use. Asthma diaries were collected at subsequent visits that occurred every 4 weeks. There were a total of six monthly follow-up visits. Our analysis focused on 161 children aged 6–11 years who met eligibility criteria. We excluded 102 participants aged 12 years or older. Figure 1 shows the details of participant flow during the study.

#### Measures

##### C-ACT

The C-ACT is a seven-item questionnaire that was developed and validated to assess asthma control among children 4–11 years old.^4^ Four questions are answered by the child and three by the parent or caregiver. The four child-reported items are scored on a 4-point numeric scale from 0 to 3 with higher numbers indicating better asthma control. One question asks, ‘How is your asthma today.’ The remaining 3 ask about activity limitation, cough and nighttime awakenings without a clear recall period. The 3 parent-reported items are scored on a 6-point numeric scale from 0 to 5, with higher numbers indicating better asthma control. Parents are asked about daytime symptoms, wheeze, and nighttime awakenings. The adult items are based on a recall period of 4 weeks. The final score of the C-ACT is the simple sum of each item and ranges from 0 to 27. Higher scores indicate better asthma control.

##### C-ACTc

The C-ACTc is comprised of only the original 4 child-reported items. The score ranges from 0 to 12 with higher scores indicating better asthma control.

##### ACQ-6

The ACQ-6 is well validated for assessing asthma control among patients older than 6 years.^24^ Items ask about nighttime awakenings, morning symptoms, activity limitations, breathlessness, wheezing and short-acting bronchodilator use. The ACQ-6 has a recall period of one week. The scores range from 0 to 6, and higher scores indicate worse control.

### ASUI

The ASUI is a 10-item questionnaire, with a 2-week recall period, designed to assess the frequency and severity of four asthma symptoms (cough, wheeze, dyspnoea and awakening at night) and side effects of asthma medications.^25^ The scores range from 0 to 1 with higher scores indicating fewer asthma symptoms.

### pAQLQ

The pAQLQ is a 23-item questionnaire that measures asthma-related functional impairments in patients 7–17 years old and comprises three domains: symptoms; activity limitation and emotional function.^26^ The pAQLQ has a recall period of one week. The scores range from 1 to 7, and higher scores indicate better asthma control.

### Episodes of poor asthma control

Participants or their parents (for those ages 6–10 years) kept daily asthma diaries documenting asthma symptom scores, nocturnal asthma awakenings, frequency of β-agonist use, morning peak expiratory flow rate (PEFR), asthma treatments and asthma-related healthcare use. Using information obtained from asthma diaries, patients were monitored for the occurrence of EPACs. An EPAC was defined as the occurrence of at least one of the following events: an increase in rescue medication use over baseline (either ⩾4 additional puffs of bronchodilator for asthma symptoms or ⩾2 additional nebuliser treatments in 1 day), the occurrence of an unscheduled contact with a healthcare provider for asthma, use of systemic corticosteroids for asthma, or a decrease of 30% or more in morning PEFR on two consecutive days, as compared with the patient’s best PEFR during the run-in period.^13^

### Spirometry

Pre- and post-bronchodilator spirometry was obtained according to American Thoracic Society (ATS) standards using the KoKo spirometer.^27^

### Statistical analysis

All statistical analyses were conducted for the C-ACT and the C-ACTc separately. Asthma diary card data were used to evaluate the patient’s asthma control for the period between visits. The C-ACT and C-ACTc scores at the end of the period or the change in scores between visits were used as dependent variables in each case. For analyses that included multiple measurements from patients, generalised estimating equations (GEE) were used to account for the correlation between repeated measures from the same patient.^28^ To evaluate the internal consistency reliability, the Cronbach *α* coefficients were calculated using baseline C-ACT and C-ACTc scores. The Cronbach’s *α* coefficient measures the degree to which items on a questionnaire measure the same unidirectional construct. To assess the test–retest reliability, we calculated the intra-class correlation coefficient (ICC) of questionnaire scores between consecutive visits, four weeks apart among patients with stable asthma. Stable asthma was defined by the absence of an EPAC between 2 consecutive visits. Construct validity was assessed by computing Spearman’s rank correlations between C-ACT and C-ACTc scores at the baseline visit with ACQ, ASUI and pAQLQ scores. The ability of the C-ACT and C-ACTc to discriminate between patient groups with different levels of asthma control was assessed by comparing mean C-ACT and C-ACTc scores across groups with or without an EPAC or an EPAC component in the prior period. Analysis of variance with adjustment for repeated measures was used to compare mean C-ACT and C-ACTc scores between the patient groups. On the basis of the occurrence of EPACS during follow-up, we defined four levels of asthma control: *good control*, no events between visits; *worsening control*, good control in the previous 4-week period followed by an event during the 4-week period before the next visit; *improved control*, an event in the previous 4-week period (prior period) but no event in the subsequent 4-week period; and *continuing poor control*, an event in the prior period and a subsequent 4-week period.^13^ Responsiveness of the C-ACT and C-ACTc was assessed by comparing the mean changes in scores from beginning to the end of each period, across the 4 levels of control using analysis of variance with adjustment for repeated measures. Mean C-ACT and C-ACTc scores were compared by means of analysis of variance between patient groups who did or did not have an EPAC.

We used both the distribution- and anchor-based methods to determine the MID for the C-ACT and the C-ACTc.^29–33^ For the distribution-based approach, baseline data were used to determine the s.d. and the standard error of the mean (s.e.m.) of the C-ACT and the C-ACTc. The s.e.m. was computed using the formula below where reliability corresponded to the Cronbach *α* coefficient for the C-ACT and C-ACTc.

$SEM=SD_{C-ACT}\times \sqrt{(1-reliability_{C-ACT})}$, $SEM=SD_{C-ACTc}\times \sqrt{\left(1-reliability_{C-ACTc}\right)}$.

By convention, the MID is calculated as 0.5×s.d.^14^ or 1×s.e.m.^15^ For the anchor-based approach, the 4 components that constituted an EPAC were used as anchors for determining change in asthma control. The mean C-ACT and C-ACTc scores between visits with an EPAC in the prior period and those without an EPAC in the prior period were calculated using GEE methods. The difference in mean scores was computed; the arithmetic average of these differences corresponded to the MID. Data were analysed using SAS version 9.0 (SAS Institute Inc.).

## Figures and Tables

**Table 1:** Patient characteristics at baseline (*n*=161)

Age at randomisation, year (s.d.)	9 (1.6)
	
*Gender, no. (%)*	
Female	59 (37)
Male	102 (63)
	
*Race or ethnic group—no. (%)*	
White	45 (28)
Black	80 (50)
Hispanic	29 (18)
Other	7 (4)
	
*Asthma characteristics*	
Age at asthma onset, year (s.d.)	2.9 (2.5)
Unscheduled healthcare use for asthma in past year, no. (%)	131 (81)
Oral corticosteroids for asthma in past year, no. (%)	119 (74)
Use of rescue inhaler ⩾2 times/week^a^, no. (%)	110 (68)
Daily use of ICS/LABA in past 6 months, no. (%)	82 (51)
Daily use of leukotriene-modifying agent, no. (%)	94 (58)
	
*Self-reported atopic conditions, no. (%)*	
Rhinitis	81 (50)
Eczema	74 (46)
Food allergies	38 (24)
	
*Asthma Questionnaires, mean (s.d.)*	
ACQ^b^	1.2 (0.8)
C-ACT^c^	19.8 (4.1)
C-ACTc^d^	8.2 (2.2)
ASUI^e^	0.82 (0.15)
pAQLQ^f^	5.4 (1.2)
	
*Lung function, mean (s.d.)*	
Per cent of predicted Pre-bronchodilator FEV_1_	94.2 (17.2)
Per cent of predicted Post-bronchodilator FEV_1_	102.2 (15.9)
Per cent of predicted Pre-bronchodilator FVC	101.3 (15.3)
Per cent of predicted Post-bronchodilator FVC	103.9 (15.5)
Change in FEV_1_ after bronchodilator	9.7 (12)
Change in FVC after bronchodilator	3.1 (7.4)

Abbreviations: ACQ, Asthma Control Questionnaire; ASUI, Asthma Symptom Utility Index; C-ACT, childhood Asthma-Control Test; FEV_1_, forced expiratory volume in the first second; FVC, forced vital capacity; ICS/LABA, inhaled corticosteroid/long acting β agonist; pAQLQ, Pediatric Asthma Quality of Life Questionnaire.

a Self-report of average use in the month before the screening visit.

b The range of ACQ scores is 0 to 6, with lower scores indicating better asthma.

c The range of C-ACT scores is 0 to 27, with higher scores indicating better asthma control.

d The range of C-ACTc scores is 0 to 12, with higher scores indicating better asthma control.

e The range of ASUI scores is 0 to 1, with higher scores indicating fewer asthma symptoms.

f The range of pAQLQ scores is 1 to 7, with higher scores indicating better asthma control.

**Table 2:** ICCs for C-ACT and C-ACTc scores between consecutive visits for stable patients

*Visit periods—4 weeks apart*	*C-ACT*	*C-ACTc*
	*ICC (*n)	*ICC (*n)
0 to 4 weeks	0.44 (26)	0.54 (26)
4 to 8 weeks	0.56 (25)	0.37 (25)
8 to 12 weeks	0.73 (23)	0.52 (22)
12 to 16 weeks	0.70 (22)	0.78 (22)
16 to 20 weeks	0.94 (18)	0.88 (18)
20 to 24 weeks	0.93 (17)	0.88 (17)

Abbreviation: C-ACT, childhood Asthma-Control Test.

**Table 3:** Spearman’s correlations C-ACT and C-ACTc to other asthma questionnaires

	*C-ACT*	*C-ACTc*
	*Spearman correlation coefficient (95% CI)*^a^	*Spearman correlation coefficient (95% CI)*^a^
ACQ	−0.56 (−0.66, −0.44)	−0.46 (−0.58, −0.33)
ASUI	0.64 (0.54, 0.72)	0.47 (0.34, 0.58)
pAQLQ	0.63 (0.52, 0.71)	0.61 (0.50, 0.70)

Abbreviations: ACQ, Asthma Control Questionnaire; ASUI, Asthma Symptom Utility Index; C-ACT, childhood Asthma-Control Test; CI, confidence interval; pAQLQ, Pediatric Asthma Quality of Life Questionnaire.

a All correlation coefficients were statistically significant at a *P* value of <0.001.

**Table 4:** Mean C-ACT scores and mean differences in C-ACT scores between patients with and without an EPAC in the prior period

*Clinical event*	*No. of EPACs/% visits*	*EPAC*	*No EPAC*	*Differences in C-ACT score*	
		*C-ACT mean (95% CI)*	*C-ACT mean (95% CI)*	*Mean (95% CI)*	P*-value*^a^
EPAC	353/46	20.2 (19.5, 20.8)	21.7 (21.2, 22.3)	1.6 (1.0, 2.0)	<0.001
					
*EPAC components*					
Decrease in PEFR	263/34	20.3 (19.6, 21.0)	21.4 (20.8, 21.9)	1.0 (0.4, 1.6)	<0.01
Increase in rescue medication use	169/22	18.9 (18.2, 19.6)	21.6 (21.1, 22.2)	2.73 (2.2, 3.3)	<0.001
Systemic corticosteroids	80/10	18.7 (17.9, 19.6)	21.3 (20.7, 21.8)	2.55 (1.8, 3.4)	<0.001
Urgent care^b^	53/7	18.5 (17.4, 19.6)	21.2 (20.6, 21.7)	2.7 (1.6, 3.8)	<0.001

Abbreviations: CI, confidence interval; C-ACT, childhood Asthma-Control Test; EPAC, episodes of poor asthma control; PEFR, peak expiratory flow rate.

a *P* values are based on linear regression models of the effect of the occurrence of an EPAC on the change in score at the next visit and accounted for correlation among repeated measures.

b Urgent care is defined as an ‘urgent unscheduled healthcare contact for asthma’ and includes emergency department, hospital, clinic or doctor’s office visits.

**Table 5:** Mean C-ACTc scores and mean differences in C-ACTc scores between patients with and without an EPAC in the prior period of 4 weeks

*Clinical event*	*No. of EPACs/% visits*	*EPAC*	*No EPAC*	*Differences in C-ACTc score*	
		*C-ACTc Mean (95% CI)*	*C-ACTc Mean (95% CI)*	*Mean (95% CI)*	P*-value*^a^
EPAC	353/46	8.6 (8.3, 8.8)	9.0 (8.7, 9.3)	0.38 (0.14,0.63)	<0.01
					
*EPAC components*					
Decrease in PEFR	263/34	8.8 (8.5, 9.1)	8.9 (8.6, 9.2)	0.08 (−0.20, 0.36)	0.57
Increase in rescue medication use	169/22	8.2 (7.8, 8.6)	9.0 (8.8, 9.3)	0.85 (0.52, 1.18)	<0.001
Systemic corticosteroids	80/10	8.2 (7.8, 8.7)	8.9 (8.6, 9.2)	0.68 (0.29, 1.07)	<0.01
Urgent care^b^	53/7	8.3 (7.7, 8.8)	8.9 (8.6, 9.2)	0.64 (0.13, 1.15)	0.03

Abbreviations: CI, confidence interval; C-ACT, childhood Asthma-Control Test; EPAC, episodes of poor asthma control; PEFR, peak expiratory flow rate.

a *P* values are based on linear regression models of the effect of the occurrence of an EPAC on the change in score at the next visit and accounted for correlation among repeated measures.

b Urgent care is defined as an ‘urgent unscheduled healthcare contact for asthma’ and includes emergency department, hospital, clinic, or doctor’s office visits.

**Table 6:** Mean change in C-ACT scores between consecutive visits by asthma control status

*Status*	N	*Visit periods*	*Mean change in C-ACT (95% CI)*
Good control	94	229	0.28 (−0.11 to 0.66)
Worsening control	79	93	−0.72 (−1.54 to 0.10)
Improved control	83	102	1.48 (0.74 to 2.22)
Continuing poor control	79	184	−0.20 (−0.77 to 0.37)

Good control—those with no events between visits.

Worsening control—those who were in good control and then had an event before the next visit.

Improved control—those with an event in the prior period but no events in the subsequent period.

Continuing poor control—those with an event in the prior period and another event in the subsequent period.

Abbreviations: CI, confidence interval; C-ACT, childhood Asthma-Control Test; *N*, number of visits.
