# Supplementary material for: Measurement characteristics of the childhood Asthma-Control Test and a shortened, child-only version
Source: NPJ Prim Care Respir Med. 2016 Oct 20;26:16075–. doi: 10.1038/npjpcrm.2016.75 (PMC5072391; doi:10.1038/npjpcrm.2016.75)
Supplement: Supplementary Table 1 [file npjpcrm201675-s1.doc]

**Supplemental Table 1**: Mean change in C-ACTc scores between consecutive visits by asthma control status

| **Status** | **N** | **Visit periods** | **Mean change in C-ACTc**  **(95% CI)** |
| --- | --- | --- | --- |
| **Good control** | 94 | 229 | 0.11  (-0.14 to 0.36) |
| **Worsening control** | 79 | 93 | - 0.17  (-0.57 to 0.22) |
| **Improved control** | 83 | 102 | 0.28  (-0.09 to 0.66) |
| **Continuing poor control** | 79 | 184 | - 0.05  (-0.35 to 0.24) |

*N – Number of visits*

*Good control* = those with no events between visits

*Worsening control* = those who were in good control and then had an event before the next visit

*Improved control* = those with an event in the prior period but no events in the subsequent period

*Continuing poor control* = those with an event in the prior period and another event in the subsequent period
